# Antibiotics, Resistome and Resistance Mechanisms: A Bacterial Perspective

**DOI:** 10.3389/fmicb.2018.02066

**Published:** 2018-09-21

**Authors:** Insha Sultan, Safikur Rahman, Arif Tasleem Jan, Mohammad Tahir Siddiqui, Aftab Hossain Mondal, Qazi Mohd Rizwanul Haq

**Affiliations:** ^1^Department of Biosciences, Jamia Millia Islamia, New Delhi, India; ^2^Department of Medical Biotechnology, Yeungnam University, Gyeongsan, South Korea; ^3^School of Biosciences and Biotechnology, Baba Ghulam Shah Badshah University, Rajouri, India

**Keywords:** antibiotics, bacteria, bacterial resistance, diseases, health care

## Abstract

History of mankind is regarded as struggle against infectious diseases. Rather than observing the withering away of bacterial diseases, antibiotic resistance has emerged as a serious global health concern. Medium of antibiotic resistance in bacteria varies greatly and comprises of target protection, target substitution, antibiotic detoxification and block of intracellular antibiotic accumulation. Further aggravation to prevailing situation arose on observing bacteria gradually becoming resistant to different classes of antibiotics through acquisition of resistance genes from same and different genera of bacteria. Attributing bacteria with feature of better adaptability, dispersal of antibiotic resistance genes to minimize effects of antibiotics by various means including horizontal gene transfer (conjugation, transformation, and transduction), Mobile genetic elements (plasmids, transposons, insertion sequences, integrons, and integrative-conjugative elements) and bacterial toxin-antitoxin system led to speedy bloom of antibiotic resistance amongst bacteria. Proficiency of bacteria to obtain resistance genes generated an unpleasant situation; a grave, but a lot unacknowledged, feature of resistance gene transfer.

## Introduction

Antibiotics, representing both naturally as well as chemically synthesized entities, emerged as a powerful tool in counteracting infectious diseases, following serendipitous discovery of penicillin from *Penicillium notatum* by Alexander Fleming in 1928. Widespread usage of antibiotics that imposes strong selection pressure for resistance development (ability to withstand effects of antibiotics) took a strong grip over the health care system globally as concerns regarding resistance to available drug regime restrict therapeutic options available to treat the disease. Emergence of resistance at rapid pace made the pathogens well-fit and well-adapted, resulting in causing serious life threatening complications as we lack robust drugs to curb the menace of multidrug resistance. Growing menace of antibiotic resistance is inevitable fallout of the introduction of new antibiotics aimed at long-term efficacy in the treatment of infectious diseases. Deteriorating public health ensuing emergence among pathogenic and commensal bacteria of resistance, illustrates a grave predicament globally (Bennett, 2008). Steadily increase in the development of resistance among bacteria thwarts current treatment regimes in hospitals and community settings. Through each passing day, treatments of infectious diseases require administration of high doses of antibiotics and longer stay in hospital. Widening gap between lean productions of drugs increases need of either to rejuvenate the drying antibacterial pipelines or design innovative strategies to combat bacterial antibiotic resistance. The present review analyses development of resistance and focusing on the factors that regulate acquisition of resistant determinants.

## Antibiotics and bacterial resistance

Antibiotics are the agents used commonly in the treatment and prevention of infections. Owing to their structure and degree of affinity to target sites, they are classified into Penicillin's, Cephalosporins, Tetracyclines, Aminoglycosides, Macrolides, Sulfonamides, Quinolones, Diaminopyrimidines, Polymyxin and Carbapenems (Sengupta et al., 2013; Bi et al., 2015; Liu et al., 2016). Being specific in their effect toward different bacterial species, antibiotics culminates them either by: (i) affecting cell wall synthesis (β-lactams), (ii) by targeting protein synthesis machinery via, interaction with ribosomal subunits (Tetracycline, Chloromphenicol, Aminoglycosides etc), (iii) disrupting with nucleic acid machinery (Rifampcin, Fluoroquinolones), (iv) interfering with metabolic pathways (Folic acid analogs, sulfonamides), and (v) by disrupting bacterial membrane structure (Polymyxins; Walsh, 2010; Table 1).

**Table 1:** Antibiotics, their mode of action and resistance mechanisms.

**S. No**	**Antibiotic (category/source)**	**Structure**	**Modification**	**Members**	**Biological effect/Mode of action**	**Resistance type**	**References**
1.	Aminoglycosides	Consist of amino-sugars connected through glycosidic bonds typically to a 2deoxystreptamine (2-DOS) core 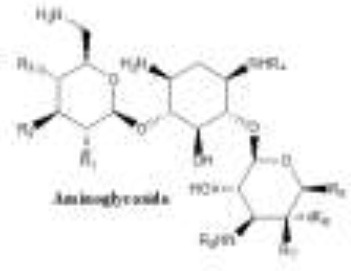	Amikacin is a semi-synthetic designer derivative of kanamycin A. The L-hydroxyaminobutyramide (HABA) side chain of amikacin blocks many AAC and APH enzymes, which increases its spectrum of activity considerably Plazomicin, semisynthetic Several modifications, including a HABA side chain, to make it resistant to almost all AMEs (aminoglycoside modifying enzymes) and have lower toxicity than other aminoglycosides.	Amikacin arbekacin, gentamicin netilmicin, tobramycin streptomycin	Inhibition of protein and cell membrane synthesis	Modification of enzymes AAC (acetyltransferases), ANT (nucleotidyl transferases or adenyl transferases), APH (phosphotransferases	Richard and Yitzhak, 2014 Jacob and Gaynes, 2010 Goossens et al., 2005
2.	β-lactams	β-lactam ring (penicillin structure) 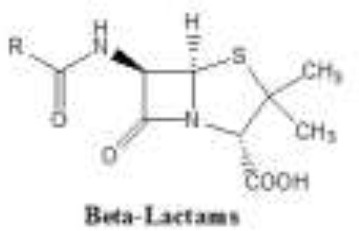	Semisynthetic methicillin and oxacillin attachment of bulky side chains increase stability toward penicillinases Other modifications were made to increase spectrum activity this include the aminopenicillins such as ampicillin and amoxicillin and ureidopenicillins like piperacillin. Carbapenems differ from other β-lactam antibiotics in that they possess a carbon instead of a sulfone in the fourth position of the thyazolidinic moiety of the β-lactam ring Clavulanic acid discovered in 1976, was the first identified β-lactamase inhibitor (Augmentin, a combination therapy of clavulanic acid and amoxicillin).	Cephalosporins, carbapenems, monobactams, β-lactam inhibitors	Interference with cell wall synthesis,	Production of β-lactamases, like extended-spectrum β-lactamases (ESBLs), plasmid-mediated AmpC enzymes, and carbapenem-hydrolyzing β-lactamases (carbapenemases), and through production of ESBL genes (bla ctx-m, bla tem, bla shv)	Bonfiglio et al., 2002 Shlaes, 2010 Spellberg et al., 2008 Projan, 2003
3.	Chloromphenicol	It is made of nitrobenzene ring consisting of nitro and dichloroacetyl group. 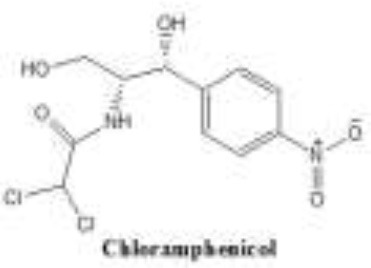	Cl_2_ group replaced with azide, nitro (NO_2_), fluorine (F) and hydroxyl (OH)	Azidamphenicol, thiamphenicol	Inhibition of protein synthesis	Enzymatic inactivation via, acetylation mediated by chloramphenicol acetyltransferases (CATs) Additional effects include inactivation of phosphotransferas, target site mutation, permeability barriers, efflux pumps	Schwarz et al., 2004
4.	Glycopeptide	Macrocyclic peptides with interspersed bridged aromatic moieties and saccharide side chains linked through glycosidic bonds. 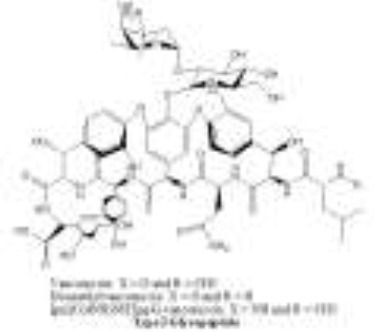	Glycopeptides differ in the amino acids at positions 1 and 3 and in the substituents of the aromatic amino acid residues. In particular, some of the carbons of the aromatic residues carry chlorine, hydroxyl or methyl groups, and some of the hydroxyl groups are substituted with sugars or aminosugars, teicoplanins, have the amino group of an aminosugar substituted with a fatty acid chain containing 9–11 carbon atoms. It is this substituent that confers greater hydrophobicity to the teicoplanin than to the vancomycin molecule.	Teicoplanin, vancomycin.	Peptidoglycan units	Inhibit cell wall biosynthesis in gram-positive bacteria by binding the terminal D-Ala-D-Ala dipeptide of peptidoglycan units sterically inhibiting their use as substrates for PBPs and transglycosylases. Five vancomycin resistant phenotypes (VanA-E), originating in VRE(vancomycin resistant enterobacteriace) Efflux mediated resistance AcrF efflux pumps have been known to cause resistance.	Reynolds, 1989 Kluytmans et al., 1997 Lina et al., 1999
5.	Quinolone	Quinolone antibiotics possess a quinolone core that typically has a N linked cyclic moiety and various substituents at the C(6) and/or C(7) positions. 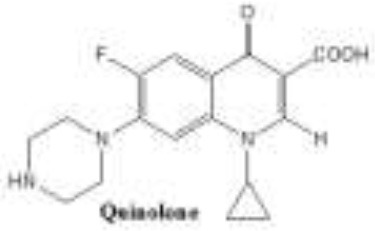	The most critical changes to the quinolone skeleton were the introduction of a fluorine at position C6 and a major ring substituent (piperazine or methyl-piperazine) at C7.1, 2, 4, 17 Because of the inclusion of the fluorine, quinolones are often termed fluoroquinolones	Cinoxacin, nalidixic acid pipemidic acid, ciprofloxacin ( enoxacin, gatifloxacin, gemifloxacin, levofloxacin, lomefloxacin, moxifloxacin, norfloxacin, ofloxacin, sparfloxacin	Topoisomerase II and I	Resistance by target modification commonly occurs by mutations to genes gyrA and parC plasmid mediated qnrA gene. Number of other Qnr proteins have also been identified in gram-negative bacteria. Fluoroquinolone efflux pumps, which can be intrinsic or acquired, commonly show broad activity against multiple antibiotic classes	Aldred et al., 2014 Emmerson and Jones, 2003 Mitscher, 2005 Andriole, 2005
6.	Sulfonamide	Have an aryl sulfonamide moiety in common 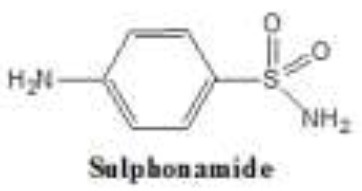			Inhibit dihydropteroate synthetase, an enzyme totally absent human cells used in folic acid metabolism. Inhibition of this enzyme ultimately leads to repressed DNA replication and bacteriostatic activity against aerobic gram-positive and negative bacteria		Richard and Yitzhak, 2014
7.	Tetracycline	Shares a common octahydrotetracene skeleton 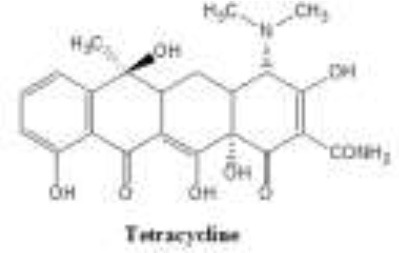		Tigecycline	30S ribosomal subunit	Tetracycline resistance is most often due to ABC efflux pumps or by ribosomal modification. A tetracycline inactivating enzyme, TetX, has also been reported.	Schaack et al., 2010
8.	Carbepenems	Carbapenems together with β-lactam ring 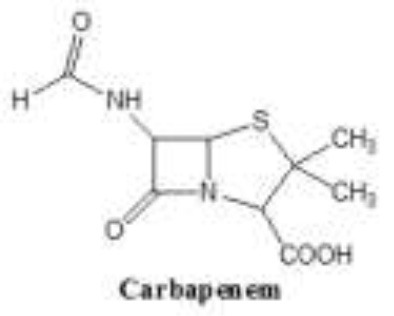		Ertapenem, faropenem, imipenem, meropenem	Penicillin binding proteins	Resistance to carbapenems in some species is intrinsic. Like in metallo-beta-lactamase (MBL) L1. In clinically important bacteria carbapenem resistance is acquired by mutational events or gene acquisition via horizontal gene transfer. Tripartite efflux pump over expression of efflux pumps Enzyme-mediated resistance (β-lactamases, carbapenemases).	Meletis, 2016 Sánchez, 2015
9.	Colistin	Cyclic heptapeptide with a tripeptide side chain acylated by a fatty acid at amino terminus. 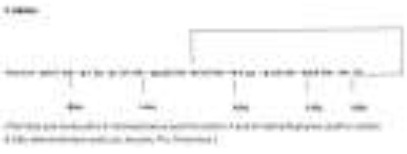	Polymyxin B and polymyxin E share almost identical primary sequence with major difference present at position 6 where D-Phe (D-phenylalanine) in polymyxin B is replaced by D-Leu (D-leucine) in polymyxin E.	Polymxin B Polymixin E (colistin)	LPS (lipopolysaccahride layer of bacteria).	Polymyxins, which are polycationic, displace stabilizing magnesium and calcium ions to electrostatically interact with the anionic lipopolysaccharide (LPS) outer layer of gram negative cell membranes. This disrupting interaction leads to increased cell membrane permeability, cell leakage, and rapid cell death.	(Yu et al., 2015) Biswas et al., 2012 Velkov et al., 2010 Liu et al., 2016

## Resistance mechanisms

### Aminoglycosides

The main aminoglycoside resistance mechanism involves modification of the enzymes. Three major classes of proteins are classified in accordance with the kind of modification: AAC (acetyltransferases) which are AAC(1), AAC(2), AAC(3), and AAC(6); ANT (nucleotidyl transferases or adenyl transferases), which includes five nucleotidyl transferases: ANT(2), ANT(3), ANT(4), ANT(6), and ANT(9), and APH (phosphotransferases) which includes seven phosphotransferases: APH(2), APH(3), APH(3), APH(4), APH(6), APH(7), and APH(9) (Kotra et al., 2000; Ramirez and Tolmansky, 2010).

### β-lactam

Resistance is acquired through production of β-lactamases, like extended-spectrum β-lactamases (ESBLs), ESBL genes (*bla*CTX-M, *bla*SHV, *bla*TEM) plasmid-mediated AmpC enzymes, and carbapenem-hydrolyzing β-lactamases (carbapenemases). Though, *Stenotrophomonas maltophilia* have endogenous metallo β-lactamases (MBL) L1 that makes it resistant to carbapenems (Sánchez, 2015). Carbapenem resistance among gram positive bacteria is acquired by mutations in the penicillin binding proteins (PBPs). However, in gram negative bacteria, lower penetration of the drug through decrease in the expression of outer membrane porin proteins such as OprD of *Pseudomonas aeruginosa* (Bonomo and Szabo, 2006). A tripartite efflux pump that causes exclusion of carbapenems from periplasmic space, adds to carbapenems resistance (Schweizer, 2003). Additionally, carbapenemases also contributes to carbapenem resistance (Poirel et al., 2007; Walsh, 2010). The main efficient carbapenemases responsible for carbapenem hydrolysis and its geographical dissemination are KPC, VIM, IMP, NDM, and OXA-48 types (Poirel et al., 2010; Nordmann et al., 2012). In a plasmid of *K. pneumoniae* HS11286 strain it was seen that deletion of *bla*_KPC−2_ abolished resistance toward carbapenem (cefoxitin, ceftazidime), and exhibited dose-dependent susceptibility toward cefepime supporting that *bla*_KPC−2_ is a key factor for the resistance toward cephalosporins and carbapenems in *K. pneumoniae* (Bi et al., 2015).

### Chloramphenicol

It acts as a broad spectrum antibiotic resistance mechanism for chloramphenicol involves enzymatic inactivation via acetylation mediated by chloramphenicol acetyltransferases (CATs) (Schwarz et al., 2004; Wright, 2005). Apart from enzyme inactivation chloramphenicol resistance mechanisms, also involves inactivation by phosphotransferases, target site mutation, permeability barriers and efflux pumps (Schwarz et al., 2004).

### Glycopeptide

The vancomycin resistance originated from the production of modified peptidoglycan precursor, d-Ala–d-Lac (VanA, VanB, and VanD) or d-Ala–d-Ser (VanC, VanE, and VanG), to which glycopeptides display diminished binding affinities. The vanA and vanB operons are positioned on plasmids as well as on chromosome; whereas the vanC1, vanC2/3, vanD, vanE, and vanG solely show their presence on chromosomes (Klare et al., 2003; Depardieu et al., 2007).

### Quinolone

Though resistance mechanism for quinolone was found restricted to chromosomes, three plasmid-mediated resistance mechanisms have also been reported (Courvalin, 2008; Martinez-Martinez et al., 2008). The chromosome-encoded resistance produce a declined outer-membrane permeability linked with porin loss, while over expression of the naturally existing efflux pumps create mutations in the molecular targets, DNA gyrase and topoisomerase IV (Hooper, 2000; Jacoby, 2005). Mutations were found occurring at quinolone resistance determining regions (QRDR) in the genes gyrA, gyrB, parC, and parE; which program the subunits of DNA gyrase and topoisomerase IV. Despite the fact that qnr determinant is the first recognized plasmid-mediated quinolone resistance gene, five new lineage of qnr genes have been accounted: qnrA, qnrB, qnrC, qnrD, and qnrS. Second kind of plasmid positioned quinolone resistant gene is a cr variant of aac(6)-Ib, that is aac(6)-Ib-cr, encoding aminoglycoside acetyl transferase (Park et al., 2006; Strahilevitz et al., 2009). The third means of resistance involves qepA, a plasmid-mediated efflux pump along with its *E. coli* derivative QepA2 (Cattoir et al., 2008), is able to expel hydrophilic fluoroquinolones, e.g., ciprofloxacin (Perichon et al., 2007).

### Sulfonamide

Sulfonamide resistance in chromosome appears through mutations in the folP gene, encoding dihydropteroate synthase (DHPS; Grape, 2006). Acquired sulfonamide resistance was identified in the 1960s, and the plasmid-mediated genes sul1 and sul2 were described after 1980s (Swedberg and Sköld, 1983; Rådström and Swedberg, 1988). In addition a third plamid mediated gene sul3 has also been recognized (Perreten and Boerlin, 2003).

### Tetracycline

Mechanisms of resistance for tetracycline hold three key strategies: energy-dependent efflux pumps (ABC efflux pumps) ribosomal protection proteins {RPPs, Tet(O)} or enzymatic inactivation (TetX; Roberts, 2002).

### Colistin

Modifications in the *lpxA, lpxC*, and *lpxD* genes of *A. baumannii* result in neutralization of lipid A biosynthesis, causing total loss of LPS leading to a loss of the polymyxin target (Moffatt et al., 2010). Polymyxin resistance is controlled by two-component systems PhoP/PhoQ and PmrA/PmrB (Olaitan et al., 2014), which react to cation (calcium, iron, and magnesium) concentrations and pH variations. These systems are concerned in the alterations of LPS resulting in polymyxin resistance. Several molecular mechanisms have been associated with colistin resistance in Gram-negative bacteria, like modifications with PmrA/PmrB, PhoP/PhoQ, ParR/ParS, ColR/ColS, and CprR/CprS two-component systems and alterations in the *mgrB* gene, that codes for negative regulator of PhoPQ. Addition of cationic groups on lipid A due to mutations creat less anionic lipid A ultimately causing less fixation of polymyxins. The polymyxins remains one of the last classes of antibiotics in which resistance is not known to spread from cell to cell via plasmid mediated. There is a current report of plasmid mediated colistin resistance in china designated as mcr-1 gene (Liu et al., 2016) which is also reported closely in five continents viz, Asia, Europe, Africa, North America, and South America (Schwarz and Johnson, 2016).

**Figure 1:**
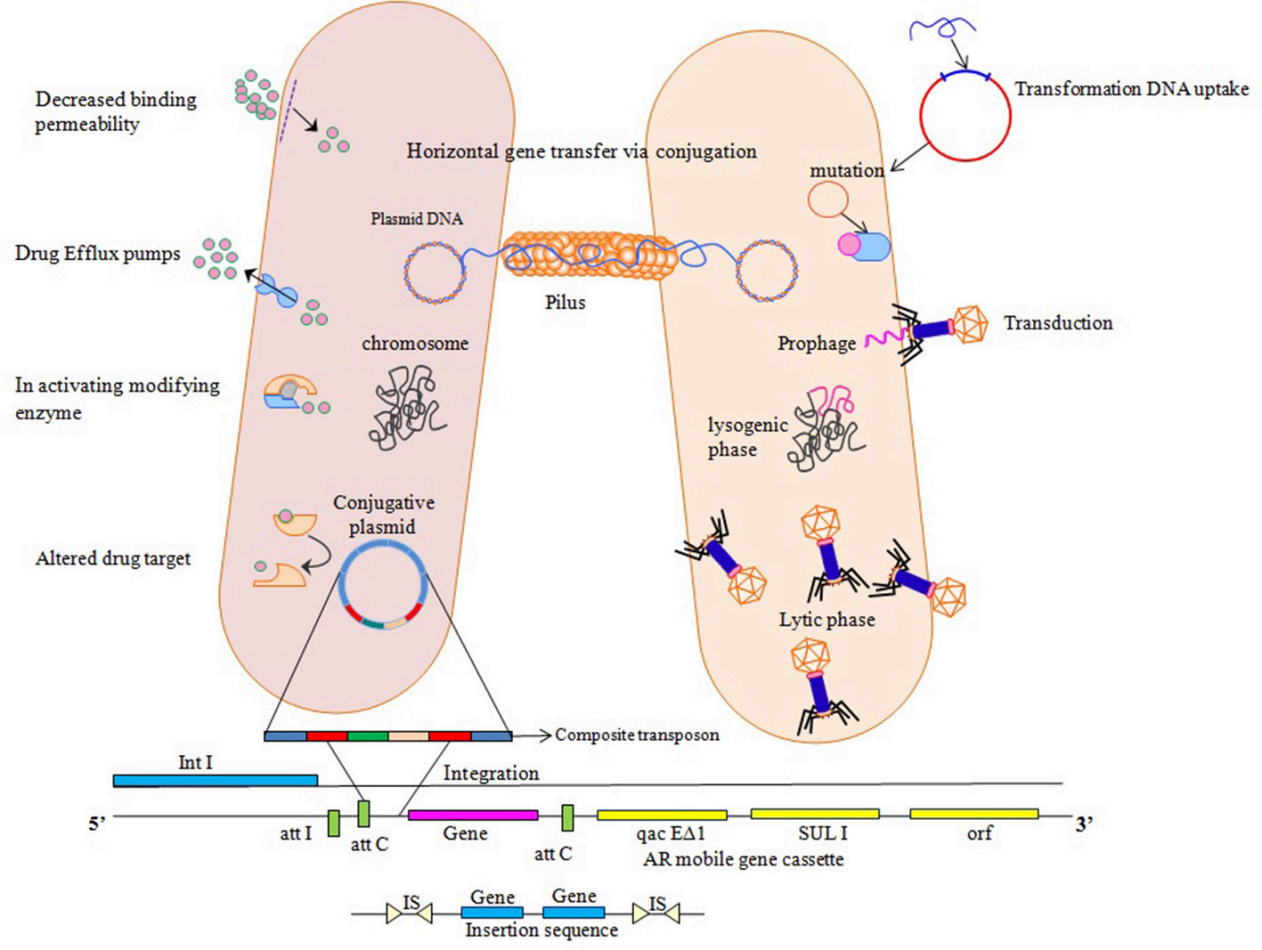
Various ways of resistance mechanisms to counteract effect of antibiotics. Horizontal Gene transfer facilitates transfer and exchange of genetic material among bacterial cells. Transformation involves direct uptake of genetic material from the surrounding by competent recipient having chromosomal set of proteins. Transduction involves DNA insertion into chromosome as prophage which then replicates, packages host DNA alone or in combination with the host cell chromosome. Conjugative plasmids utilize a protein structure pilus to make a link with the recipient cell so as to move them into the recipient cell that ultimately transfers the copy of entire bacterial chromosome, multicopy plasmid or a small portion to a recipient cell, where these genetic elements insert into the chromosome or replicate independently if compatible with the inhabitant plasmids. Integrons use site specific recombination mechanism where it provides a promoter for gene cassettes to exchange and disseminate. Transposons and insertion sequences insert into new sites on the chromosome or plasmids by non-homologous recombination and increase the copy number of transferred genes giving rise to chromosomal mutations, deletions and rearrangements.

Antibiotics do not, in themselves, cause resistance but frequent and high exposure of antibiotics to bacteria creates a selection pressure which triggers resistance strategies of bacteria. Acquirement of resistance genes has been viewed as main donor in favor of the extensive dispersal and increase in antimicrobial resistance through horizontal transfer involving MGEs (Xu et al., 2011). Their presence on mobile genetic elements facilitate transfer to un-related bacteria in a process referred to as horizontal gene transfer (HGT) via, conjugation, transduction, or transformation (Aminov and Mackie, 2007; Martinez, 2008). Transformation involves movement of cellular DNA among closely linked bacteria, imparted by chromosomal set of proteins that occur in naturally transformable bacteria. Conjugation needs autonomously replicating genetic elements known as conjugative plasmids that cause movement of plasmid from the donor cell to a recipient cell that is devoid of it. Transduction involves transfer of DNA facilitated by bacteriophages, constituted host DNA in their capsid and insert this DNA into a new host, where it combines with cellular chromosome and is inherited (Frost et al., 2005). Movement of genes confers new metabolic capabilities to the recipient, thereby helps them in their adaptation to new ecological niches. Resistance to antibiotics conferred by chromosomal or mobile genetic elements, is achieved by following strategies: (i) reduction of membrane permeability to antibiotics either by decreasing uptake or increasing efflux, (ii) drug inactivation either by hydrolysis or by modification, (iii) alteration in drug target and decreased binding permeability, and (iv) mutation (Walsh, 2000; Figure 1).

## Genetic basis of antibiotic resistance

Bacteria appeared on this planet billion of years ago, so have their skills sharpened due to genomic flexibility at shielding themselves from toxic chemicals. Bacteria are well-known potent originators for the dissemination of antimicrobial-resistant genetic apparatus (Woodford et al., 2011). They are competent to offer secured platform for the upholding and transmission of genes accountable for antimicrobial resistance as part of mobile genetic elements (MGE; plasmids, transposons and integrons). The transposons and integrons, owing to their genomic plasticity have contributed a great deal to the fitness quotient and robustness of bacteria to survive in varying environments. Integrons, typically transported by plasmids or enclosed in transposons, performing the task of resistance gene dissemination plays an important role in the revealing of Super Bugs (Xu et al., 2011). Since its earliest assessment in 1989 (Stokes and Hall, 1989) molecular mechanisms involved in the mobility of integrons, their excision and integration for gene cassettes, is currently being scrutinized (Hall et al., 1999; Mazel, 2006). Establishing role of MGEs in genomic evolution justifies the predictions of Barbara McClintok that transposons play a major role in the genomic diversity and evolution. Owing to their capacity to relocate between host genomes, MGEs play a vital function of acting as vehicles for resistance gene acquisition and their successive propagation.

### Resistance mediated by plasmids

Plasmids that mediate horizontal movement of plasmid-borne genes are accountable for global spread of resistance (Carattoli, 2013). Resistance plasmids (attributing resistance to commonly used antibiotics) are mostly conjugative; additional are mobilizable. Conjugative plasmids display both broad (no host restriction within the division) and narrow (shifting limited to small number of related bacterial groups) host range. Genes acquired through homologous recombination, integration and excision from the host chromosome relocate from donor to recipient cells by conjugation. These type of plasmid-encoded complexes help the contributor by attaching to promissing recipient that lead to the generation of secured association, required prior to the relocation of DNA. Plasmids that fails to get reloctaed by this approach are transferred by conjugative elements subsequent to the development of transitory or steady fusions called cointegrates. Plasmids also encourage cell contact development through production of pheromone influenced microfibrillar exterior covering substances. Mobilizable plasmids carry DNA transfer genes essential for structure of all or element of the relaxasome, but are deficient of genes essential for mating pore formation. They have a capacity to use conjugative plasmids for horizontal spread; these are immobile in cells which are short of mobile elements carrying compatible mating-pore genes. The majority of known mobilizable plasmids utilize conjugative element mating-pores by expressing their own relaxase (Mob) which works on the plasmid's cognate oriT (Joshua et al., 2016). Current studies have revealed that plasmid transfer can also take place even when the mobilizing plasmid and the plasmid being mobilized are in two diverse bacterial cells (Andersen and Sandaa, 1994; Sia et al., 1996). This type of recruitment, in which a contributor strain possessing a self-transmissible plasmid is getting a second plasmid from a receiver strain, is known as retrotransfer. Retrotransfer take place through two stages; (1) self-transmissible plasmid move from the contributor to the receiver, and (2) mobilization of plasmid from receiver back to the contributor (Ankenbauer, 1997). As the capacity of a self-transmissible plasmid to promote the acquirement of novel plasmids by its bacterial host possibly grant a benefit to the contributor bacterium, it can be said retrotransfer may play a significant job in the progression of plasmid transfer system.

Transphylum mobilization events that incorporate elements from entirely diverse phylogenetic group of bacteria, underscores broad range of interactive capacity originated in gene relocation elements. Besides being recognized through PCR amplification of identified incompatibility groups (Götz et al., 1996), mobilizing plasmids are identified by conducting a triparental mating among *E. coli* having a mobilizable IncQ plasmid, a recipient lacking plasmid and amalgam of local soil or marine bacteria (Top et al., 1994). Endurance of plasmids in natural isolates and their perceptible firmness in absence of antibiotics, opposes a common thought that in non-existence of selection pressure plasmids additional gene transport factors are easily lost. The multitude of antibiotic-resistant strains in environmental milieu where bacteria apparently do not appear to be in touch with antibiotics, suggest that resistance genes can also be firmly retained even in the paucity of antibiotic selection (Andersen and Sandaa, 1994; McKeon et al., 1995; Calva et al., 1996). As plasmids exhibit remarkable property of crossing species borders effortlessly (Hatch and Michael, 2011) co-mobilization of resistance genes aggravates furthermore clinical crisis.

### Resistance mediated by transposons

Transposable elements (TE) are the DNA sequences that provide flexibility to the genome (Archana et al., 2013). Being proficient to alter their position, they are able to alter their genetic background along with that they change the genetic setting of the locus, where they get inserted (Wicker et al., 2007; Shapiro, 2010). Based on their role in identification and recombination of particular sequences, TEs are categorized into two classes; composite transposons (Class I; holding a range of resistance genes which possess identical structural and functional characteristics, but small DNA homology) and complex transposons (class II; constituting three dissimilar but interrelated families; Tn3, Tn21 and Tn2501; Schmitt, 1986; Wiedemann et al., 1986; Lafond et al., 1989). Some of the composite transposons in gram negative bacteria are Tn5, Tn9, Tn10, Tn903, Tn1525, and Tn2350 and among gram positive bacteria are Tn4001 and Tn4003. Compared to Tn1, Tn3, Tn21, Tn501, Tn1721, and Tn3926 found among gram negative bacteria, gram positive bacteria encompass Tn551, Tn917 and Tn4451 complex resistance transposons. These components are possessed with the capability to progress both intra and inter-molecularly which means they can jump within a DNA molecule or from one DNA molecule to another (Bennett, 2008). Tn21 being majorly studied, bear OXA (a carbapenems, possess oxacillinase activity) and PSE (β-lactam gene Pseudomonas specific enzyme) determinants that makes them resistant to aminoglycoside antibiotics. Tn21 also show resistance toward mercury compounds (Brown et al., 1986) and trimethoprim imparted by dhfr II and V (Sundström et al., 1988). Class I or retro-transposons work by copying RNA from DNA by transcription and RNA to DNA by reverse transcription; thereby get inserted into the genome at a diverse location (Kapitonov and Jurka, 2008). Acting in cut and paste manner, class II transposons does not involve RNA intermediate (Wicker et al., 2007). These, transposases create staggered cut at specific site, creating sticky ends; following its transposition to the aimed site, generally followed by target site duplication and construction of short direct repeat at insertion sites (Madigan et al., 2006). Though transposons provide antibiotic resistance due to the existence of an extra gene on a plasmid, there are chances that transposons can jump from chromosomal DNA to plasmid DNA and vice versa for development of resistance (Wagner, 2006).

Insertion Sequences (ISs; size < 2.5 kb) are basic form of mobile genetic elements disseminated in bacteria. ISs are contemplated as non-complex bacterial mobile DNA taking into account their structure (Allaaeddin El et al., 2013). They include more than 19 families, having dissimilar size (Wagner et al., 2007). ISs include an open reading frame that codes for a transposase enzyme, surrounded by inverted repeat sequences of 10–40 base pairs at both ends. The transposase enzyme cuts target DNA and inserts the IS due to possible association with the inverted repeat sequences. Exhibiting fondness toward AT-rich region of DNA, higher chances of undergoing homologous recombination, creates variety of possibilities such as deletions, inversions and duplications. There are evidences that when two identical IS elements surround a region of DNA, a composite transposon is produced, and the total interceded DNA flanked by the terminal inverted repeats get mobilized by one or both of the IS coded transposases (Ochi et al., 2009; Gyles and Boerlin, 2014).

### Resistance mediated by integrons

Integrons attribute a great deal to the fitness quotient and robustness of bacteria to survive in varying environments. Harboring resistance determinants such as antibiotic resistance genes, their mobilization as part of chromosomes and plasmids and integration far off from their origin confer resistance to antimicrobials. Their categorization is based on amino acid sequences of integrase IntI; those carrying IntI1 are referred as class 1, IntI2 as class 2, IntI3 as class 3 and so on. Integrase IntI1, IntI2, and IntI3 were found associated with mobile genetic elements, while IntI4 was found linked with chromosomal integrons.

#### Class 1 integrons

Class I integrons are found associated with the acquisition and mobilization of antibiotic resistance genes. Originated from Tn402, they are composed of two sequence; 5′ conserved sequence (5′CS) representing an integrase gene and a 3′ conserved sequence (3′CS) encoding quaternary ammonium compound resistance gene (qacΔE1) and sulfonamide resistance gene (sul1), respectively (Cambray et al., 2010). With three recombination sites (*attI1, attC* and secondary site), expression of captured gene cassettes acquired via site-specific recombination is driven by a promoter located in the 5′-conserved segment (5′-CS) region (Collis et al., 1993). Class 1 integrons are associated with a variety of resistance gene cassettes, but most integrons contain an *aadA* resistance determinant, encoding streptomycin-spectinomycin resistance. Trimethoprim resistance determinants are also detected frequently (Fluit and Schmitz, 2004; Mazel, 2006; Cambray et al., 2010). Showing prevalence of 22–59%, its localization is reported among diverse groups of Gram negative bacteria; *Escherichia, Klebsiella, Aeromonas, Enterobacter, Providencia, Mycobacterium, Burkholderia, Alcaligenes, Campylobacter, Citrobacter, Stenotrophomonas, Acinetobacter, Pseudomonas, Salmonella, Serratia, Vibrio, and Shigella* (Ramírez et al., 2005; Crowley et al., 2008; Partridge et al., 2009; Xu et al., 2009, 2011). Gram positive bacteria; *Enterococcus, Corynebacterium, Streptococcus, Brevibacterium, Aerococcus, and Staphylococcus* show high prevalence of *aadA* and *dfrA* gene cassettes (Nandi et al., 2004; Xu et al., 2010; Veise et al., 2013).

#### Class 2 integrons

Class 2 integrons associated with the Tn7 transposon family (Tn*1825*, Tn*1826*, and Tn*4132*), carry a recombination site *attI2* and promoter within these transposons (Xu et al., 2009). Its 3′ conserved segment (3′-CS) contains 5 tns genes (*tnsA, tnsB, tnsC, tnsD* and *tnsE*) associated with movement and preferential insertion at unique site within bacterial chromosomes (Hansson et al., 2002; Labbate et al., 2009). The amino-acid sequences coded by *intI2* gene show <50% homology with *intI1*, and its non-functionality was found attributed by replacement of glutamic acid with a termination codon (amino acid 179) that leads to production of a shorter and inactive polypeptide (Barlow and Gobius, 2006). The classic structure of class 2 integrons contain a range of gene cassettes, including streptothricin acetyltransferase (*sat1*), adenyltransferase (*aadA1*), dihydrofolate reductase (*dfrA1*; Hansson et al., 2002; Xu et al., 2009). Class 2 integrons have been reported among *Salmonella, Enterobacteriaceae, Acinetobacter*, and *Psuedomonas* (Machado et al., 2008; Vinué et al., 2008; Macedo-Viñas et al., 2009; Ozgumus et al., 2009; Xu et al., 2009, 2010, 2011).

#### Class 3 integrons

Class 3 integrons (*IntI3*) create excision of integrated cassettes and integration of circularized cassettes into the *attI3* site with a considerably lower recombination than that observed with *IntI1* (Arakawa et al., 1995). This class of integron was firstly identified from *Serratia marcescens* in 1993, and then found associated with *bla*GES-1 from *Klebsiella pneumoniae* strain FFUL 22K. Class 3 integron containing *bla*GES-1 within the IncQ plasmid was also found in *E. coli* (Collis et al., 2002). Occurence of class 3 integrons associated with IMP-1 metallo-beta-lactamase is limited to *Acinetobacter, Alcaligenes, Citrobacter, Escherichia, Klebsiella, Pseudomonas, Salmonella*, and *Serratia* (Arakawa et al., 1995; Rowe-Magnus et al., 1999, 2001; Ploy et al., 2003).

#### Class 4 integrons

Class 4 integrons are distinguished from other Resistance Integrons (RIs) by two key features; incorporation of hundreds of cassettes (*V. cholerae*, 216 unidentified genes in an array of 179 cassettes, holding roughly 3% of the genome) and the high similarity between the *attC* sites of those assembled cassettes (Poirel et al., 2010). Class 4 integrons carry gene cassettes for antibiotics chloramphenicol and fosfomycin (Fluit and Schmitz, 2004). Inspite of huge array of cassettes, identification of class 4 integron has been limited within members of *Pseudomonas, Xanthomonas, Shewanella, Vibrionaceae*, and other proteobacteria (Clark et al., 2000; Rowe-Magnus et al., 2001).

### Integrative and conjugative elements

Integrative and conjugative elements (ICEs) were first of all anticipated by Burrus et al. (2002) are different mobile elements found in both Gram-positive and Gram-negative bacteria. These are self transmissible integrative elements that code for complete match of conjugation apparatus. ICEs can integrate into and excise from a host chromosome. These versatile entities support their own mobilization facilitating horizontal transfer of antibiotic-resistant genes, virulence factors and various bacterial traits. ICEs possess three genetic modules: (i) integration and excision module; (ii) conjugation module; and (iii) regulation module. These modules contain different array of genes that code for proteins operating by distinct mechanisms. ICEs contain a gene encoding an integrase (Int) that promotes site-specific integration and excision of the element, frequently into a unique site on the chromosome of the host organism (Boyd et al., 2009). Some ICEs bear maintenance modules such as toxin–antitoxin systems (Wozniak and Waldor, 2009) and additional partition systems that guarantee thriving vertical inheritance of these elements. In contrast to plasmids, ICEs, are not found in extrachromosomal state, because they lack autonomous replication, the first known MGEs with ICE-like properties were Tn*916* in *Enterococcus faecalis* and CTnDOT in *Bacteriodes thetaiotaomicron*; Franke and Clewell, 1981; Shoemaker et al., 1989). Bacteroides CTnDOT promote dissemination of antibiotic-resistant genes (Whittle et al., 2002). ICEs are distinguished by element-specific properties although they possess a general life cycle and modular structure. Apart from resistance to antibiotics (Böltner et al., 2002; Whittle et al., 2002) ICEs show a extensive collection of phenotypes on their hosts, including resistance for heavy metals (Böltner et al., 2002; Davies et al., 2009) and the power to degrade aromatic compounds (Ravatn et al., 1998). In addition, complex traits such as the capacity to inhabit a eukaryotic host (Sullivan and Ronson, 1998) fix nitrogen (Sullivan and Ronson, 1998) or encourage virulence and biofilm development have been recognized (Drenkard and Ausubel, 2002; He et al., 2004; Davies et al., 2009) the connection between ICEs and the propagation of antibiotic resistance genes in some pathogens show that these mobile elements have considerable clinical significance (Hochhut et al., 2001; Whittle et al., 2002; Mohd-Zain et al., 2004). ICEberg (http://db-mml.sjtu.edu.cn/ICEberg/) is an integrated database that provides comprehensive information about integrative and conjugative elements (ICEs) found in bacteria (Bi et al., 2012).

### Bacterial toxin anti-toxin systems

Toxin-antitoxin (TA) systems, initially identified as plasmid addiction modules, are plentiful in the chromosomes of most free-living bacteria (Xie et al., 2018). TA systems provide endurance to bacterial populations in conditions of stress like nutrient deprivation or antibiotic pressure (Harms et al., 2016). Generally TA systems are made of a stable toxin and a labile antitoxin coded by a bicistronic locus (Lee and Lee, 2016). Toxin genes encode for proteins, while the antitoxin genes code for RNAs or antitoxin proteins, classifying them as type I–VI TA loci (Gerdes and Maisonneuve, 2012; Chan et al., 2016; Page and Peti, 2016) categorized due to mechanisms applied by the antitoxins to counteract the actions of the toxins. In TA systems I-VI product of the toxin gene is typically a protein, whereas the antitoxin gene is either a non-coding RNA among TA I and III or a low-molecular-weight protein in TA systems II, IV, V and VI. Toxins work on diverse targets to distress various cellular processes such as DNA replication, cell wall synthesis or protein synthesis. Amongst six types of TA system, type II is broadly studied, due to great quantity and high quality of freely accessible data. Currently two open-access bioinformatics resources in the field of type II TA loci, the online tool RASTA (Sevin and Barloy-Hubler, 2007) and the web-based database TADB (Shao et al., 2011) are available.

Presently, a new toxin is reported that contains a Gcn5-related N-acetyltransferase (GNAT) domain that transfers the acetyl group from acetyl coenzyme A (Acetyl Co~A) to the amine group of tRNAs (Jurenas et al., 2017b; Van Melderen and Wood, 2017) resulting in acetylation of tRNAs following inhibition of translation in bacterial cells. Similarly TacT of *Salmonella enterica* Typhimurium (Cheverton et al., 2016) and AtaT of *Escherichia coli* O157:H7 (Jurenas et al., 2017a), also transfer the acetyl group from acetyl coenzyme A to the amine group of the tRNAs, In stress environment, the intensity of the alarmone molecule, (p)ppGpp, is amplified, which activates a particular proteinase that degrades the antitoxin by proteolytic cleavage, thus permitting the toxin to stop cell expansion. The free toxins then cause the dormant state of bacterial cells (persister cells) which can encourage bacterial tolerance to antibiotics (Gerdes and Maisonneuve, 2012). Persisters are a fraction of bacterial cells in the culture that survive through a prolonged antibiotic treatment. Many studies have shown that toxins of diverse chromosomal TA systems encourage the development of persister cells. GNAT-RHH TA system is a newly exposed approach of bacterial cells to support persister cell formation by disturbing tRNA functions (Jurenas et al., 2017b), in *S. enterica* over expression of the TacT causes drug tolerance (Cheverton et al., 2016). Chromosomal type II TA loci have been reported to be activated by environmental stress (Li et al., 2016). To assess if antibiotic stress would stimulate the transcription of kacAT operon, exponential phase HS11286-RR2 cells were checked with different antibiotics at the minimum inhibitory concentration (MIC). Transcript levels of the toxin gene, kacT, were quantified by RT-qPCR at various time points after antibiotic challenge. RT-qPCR results showed that the exposure to meropenem or tigecycline antibiotics caused 10-fold or 40-fold increase in kacT transcription levels (Qian et al., 2018).

*Klebsiella pneumoniae* faces a wide diversity of environmental conditions, including antibiotic stress. Fifteen pairs of putative type II TA loci are detected on the *K. pneumoniae* HS11286 chromosome. Activation of the toxin plays an important role in bacterial multidrug tolerance (Harms et al., 2016). The chromosomally encoded kacAT bicistronic operon of *K. pneumoniae* HS11286 is a functional GNAT-RHH TA locus with kacA encoding the cognate antitoxin to the toxic product of kacT. (Hall et al., 2017). Over expression of KacT inhibited *K. pneumoniae* cell growth and resulted in dormant cell formation. Crystal structure analyses show that the KacT toxin adapts a typical GNAT-fold, which may confer the same catalytic mechanism as the one revealed for TacT of S. enterica (Cheverton et al., 2016). It may bind cellular tRNAs via its positive groove and transfer the acetyl group from AcCoA to tRNAs halting translation leading to cell arrest.

## Strategies to combat bacterial antibiotic resistance

Emerging antibiotic resistance is a problem of global magnitude. Confronted by increasing amounts of antibiotics use, emergence and dissemination of antibiotic resistant strains has compromised therapeutic potential of antibiotics. Of the different strategies adopted, techniques that materialize ideally include; (1) Designing antimicrobial peptides (AMPs; Bacteriocins, Cathelicidins, Microcins, etc.) with broader spectrum of targets (Gordya et al., 2017), (2) Phage therapy (exploiting phages such as OMKO1, wksl3 and Φ1 to kill antibiotic-resistant bacteria; Chan et al., 2016), (3) combination therapy (using combination of antibiotics e.g., colistin in association with tigecycline, aminoglycoside, meropenem, etc or combination of inhibitor and antibiotic such as Augmention i.e., combination of clavulanate and amoxicillin; Soudeiha et al., 2017), (4) Delivery of drugs, AMPs and essential oils as nanoparticles (NPs) for sustained and controlled release (AgNPs of penicillin G, amoxicillin, erythromycin, and vancomycin; Wang et al., 2017), (5) Liposomes as drug targeting vehicles (Poerio et al., 2017), (6) Use of natural compounds such as Flavonoids, Alkaloids, Coumarins, etc. (Chandra et al., 2017), and (7) Modification of antimicrobials e.g., Plazomicin (ACHN-490); derivative of sisomicin produced by addition of a hydroxy-aminobutyric acid substituent at position 1 and a hydroxyethyl substituent at position 6 (Lopez-Diaz et al., 2017; Table 2). Other approaches include use of genomics to find out new bacterial targets and optimization of newer approaches that target bacterial pathogens while exerting selection for reduced pathogenesis, if bacteria evolve resistance to therapeutic intervention. Additionally, strategies such as designing molecules that can block bacterial attachment to surfaces and target bacterial virulence factors along with contribution to protect through production of inactivating antibodies, seems other suitable options to over the menace of drug resistance.

**Table 2:** Strategies to combat the menace of drug resistance.

**S. No**	**Strategy**	**Entity**	**Explanation**	**Target species/effect**	**References**
1.	Antimicrobial peptides (oligopeptides with a varying number of amino acids)	Bacteriocins	Cationic and amphiphilic peptides containing 20–50 amino acids. Their interactions with negatively charged bacterial membrane lead to formation of transmembrane pores that causes leakage of cellular solutes, and eventually cell death. Genetic determinants for bacteriocin production are located on mobile genetic elements. Most bacteriocins are reported from *E. coli* and other enterobacteria.	They target pathogens including *Clostridium difficile* and emerging antibiotic-resistant bacteria such as MRSA, VRE and enterohaemorrhagic *E. coli* via, inhibition of cell wall biosynthesis. Lysostaphin bacteriocin exhibitsbactericidal activity against *Staph. aureus* and *Staph. epidermidis*.	Hassan et al., 2012 Gordya et al., 2017 Maria-Neto et al., 2015 Kościuczuk et al., 2012 Kaur et al., 2016
		Defensins	They are a group of AMPs containing α-helix/β-sheet elements coordinated by three disulfide bridges.	They are effective against Gram positive bacteria.	Lehrer et al., 1989; de Leeuw et al., 2010
		Cecropins	They are linear amphipathic α-helical AMPs	They act selectively active against Gram-negative bacteria.	Cirioni et al., 2008
		Diptericins	They are members of glycine-rich AMP family	Their selective toxicity against Gram-negative Enterobacteria like *E. coli* occurs via, disruption of cell wall.	
		Cathelicidins	They are small, cationic, antimicrobial peptides, varying in amino acid sequence, structure and size. They are stored in the secretory granules of neutrophils and macrophages, released extracellular upon leukocyte activation.	They exhibit broad spectrum of activity against bacteria, enveloped viruses and fungi. Main target is bacterial cytoplasmic membrane.	Zanetti, 2004; Kaneider et al., 2007
		Microcins	It is a low-molecular weight antimicrobial peptide produced by Gram negative Enterobacteria as host defense peptides. They are < 10 kDa in size, much smaller than other antimicrobial peptides.	They display strong antimicrobial activity against Gram-negative bacteria, such as *E. coli* O157:H7, *Salmonella enteritidis* and *S. typhimurium*. Inhibit DNA replication by targeting DNA gyrase.	Nocek et al., 2012; Rebuffat, 2012
		Auranofin's	Its ability to suppress bacterial protein synthesis leads to significant reduction in the production of key methicillin-resistant *Staphylococcus aureus* (MRSA) toxins	Inhibition of multiple biosynthetic pathways including cell wall, DNA, and bacterial protein synthesis.	Thangamani et al., 2016
		Buforin II	21 amino acid cationic and linear molecule peptide. Crosses cell membrane without permeabilizing it.	Inhibition of DNA replication and protein synthesis	Cho et al., 2009; Xiea et al., 2011
2.	Phage therapy	OMKO1, wksl3 and Φ1	A new approach to therapy where bacteriophages exert selection for MDR bacteria to become increasingly sensitive to traditional antibiotics.	Ability of phages to kill antibiotic-resistant bacteria allied with their ubiquitous nature, high specificity (minimal disruption of normal flora), self-replication ability at the infection site, and more importantly low inherent toxicity qualifies them as “safe” and “green” technology.	Chan et al., 2016
3.	Combination therapy	Antibiotic-antibiotic	Colistin in association with tigecycline, aminoglycoside, meropenem, imipenem	These antibiotic combination showed a decrease of 2.6- to 2.8-fold in MIC	Soudeiha et al., 2017 Bae et al., 2016
		Antibiotic inhibitor	Combination of inhibitor and antibiotic such as Augmention i.e., combination of clavulanate and amoxycillin)		
4.	Nanoparticle based delivery of drugs, AMPs and essential oils	Delivery of drug, AMPs and essential oils	AgNPs of penicillin G, amoxicillin, erythromycin, and vancomycin show enhanced antibacterial and anti bio-film formation in bacteria like *Acinetobacter baumannii, Enterococcus faecalis, Klebsiella pneumoniae, Pseudomonas aeruginosa, Staphylococcus aureus, Vibrio cholera* by Alteration of membrane permeability, cell wall and cytoplasm as well as by Irreversible damage on bacterial cells. Au, Mg, NO, ZnO, CuO, Fe3O4 and YF NPs also ceases biofilm formation.	Demonstrated the improvised antibacterial activity. Since nanoparticles do not enter the bacterial cell, and its mechanism of killing bacteria is fundamentally done via direct contact with the bacterial cell wall.	Beyth et al., 2015; Franci et al., 2015; Wang et al., 2017
5.	Liposomes as drug delivery vehicles	Drug loaded liposomes	Liposomes are spherical vesicles, with particle sizes ranging from 30 nm to several micrometers, consisting of one or more lipid bilayers surrounding aqueous spaces used as targeted drug delivery systems.	Liposomes like **BBLs** (biomineral-binding liposomes), **LLSs** (liposome loaded scaffolds), **SSLs** (solid supported liposomes) help in the delivery of drugs like Vancomycin, gentamicin, Triclosan, chlorhexidine, Benzyl penicillin G, Amikacin, Tobramycin, Meropenem etc. **ABL** (apoptotic body-like) resulting in the reduced biofilm formation by the bacteria like *E. coli P. aeruginosa A. baumannii, S. aureus, S. oralis*.	Nag and Awasthi, 2013; Rukavina and Vanic, 2016; Poerio et al., 2017
6.	Use of natural compounds	Flavonoids (Isocytisoside Eucalyptin)	Pigmented compounds found in fruits and flowers of plants which include flavone, flavanones, flavanols, and anthocyanidins.	They show activity against MDR *Pseudomonas aeruginosa, S. Typhi, E. coli, K. pneumoniae*. Disruption of membrane stability by increasing membrane permeability.	Chandra et al., 2017 García et al., 2012 Savoia, 2012 Zeng et al., 2010
		Alkaloids (Berberine)	Consists of heterocyclic nitrogenous compounds	Exhibit broad spectrum antimicrobial activity *P. aeruginosa, E. coli, S. aureus, S. mutans, M. gypseum, M. canis and T. rubrum*.	Savoia, 2012
		Coumarins (Asphodelin A)	They are aromatic benzopyrones with fused benzene and alpha pyrone rings	They possess activity against *S. viridians, S. mutans*, etc	García et al., 2012
7.	Modification of antimicrobials	Plazomicin (ACHN-490)	Derivative of sisomicin produced by addition of a hydroxyl-aminobutyric acid substituent at position 1 and a hydroxyethyl substituent at position 6	A bactericidal aminoglycoside with enhanced activity against MDR Gram-negative bacteria and *S. aureus*.	Tillotson and Theriault, 2013 Tsodikova and Labby, 2016 Lopez-Diaz et al., 2017

## Conclusion

Bacterial infections continue to be one of the leading causes of morbidity and mortality worldwide. Fallout of excessive and imprudent antibiotic use, widespread dissemination of resistant determinants as part of MGEs has increased the rate of resistance development. Being capable to relocate between host genomes, they act as vehicles for resistance gene acquisition and their successive propagation. Thorough molecular studies have identified several mechanisms in microbes to attain the antimicrobial resistance. Among these mechanisms, plasmids, transposons, insertion sequences, integrons, ICEs and bacterial Toxin Anti-toxin systems have exposed how and why resistance has attained alarming stage. The possibility for recombination of genes from different bacterial populations is huge and it seems that it doesn't take bacteria much time to acquire the genetic resources to flourish in surroundings that would have otherwise hindered it's growth. Occurring with increasing frequency, resistance limits therapeutic option, resulting in the cases where certain human infections cannot be treated. Pertinently, where there is stiff resistance on the implementation of evidence-based clinical practice, scientists of the health care organizations are still searching as how to keep pace with the demand of actionable knowledge. This adverse condition of antimicrobial resistance demands the rejuvenation of dried pipeline for the development of new and efficient drugs to treat the deadly infection. With a goal to get hold of the menace of antibiotic resistance, it seems essential for everybody to have some basic knowledge about the systems in order to ensure optimal use of antibiotics from the surrounding milieu, to slow down the development of antibiotic-resistant superbugs.

## Author contributions

QH and AJ conceived the idea. IS, SR and AJ contributed to writing of the manuscript. MS and AM contributed to reference and graphics section.

### Conflict of interest statement

The authors declare that the research was conducted in the absence of any commercial or financial relationships that could be construed as a potential conflict of interest.
